# Characterization and Functional Analysis of the Poplar *Pectate Lyase-Like* Gene *PtPL1-18* Reveal Its Role in the Development of Vascular Tissues

**DOI:** 10.3389/fpls.2017.01123

**Published:** 2017-06-28

**Authors:** Yun Bai, Dan Wu, Fei Liu, Yuyang Li, Peng Chen, Mengzhu Lu, Bo Zheng

**Affiliations:** ^1^Key Laboratory of Horticultural Plant Biology of Ministry of Education, College of Horticulture and Forestry Sciences, Huazhong Agricultural UniversityWuhan, China; ^2^National Key Laboratory of Crop Genetic Improvement and National Centre of Plant Gene Research, College of Plant Science and Technology, Huazhong Agricultural UniversityWuhan, China; ^3^State Key Laboratory of Tree Genetics and Breeding, Research Institute of Forestry, Chinese Academy of ForestryBeijing, China

**Keywords:** pectin, pectate lyase, cell wall, wood formation, *Populus*

## Abstract

Pectin is a major component of plant cell walls, and the structure of pectin impacts on the properties of wood. Although we know that pectate lyase (PL, EC 4.2.2.2) has a major influence on the structure of pectin, our knowledge of *Pectate lyase-like genes* (*PLL*) in tree species remains limited. To better understand the characteristics of *PLL* genes in trees and to identify novel *PLL* genes that are potentially involved in the development of wood, we performed comprehensive analyses of gene structures, phylogenetic relationships, chromosomal locations, gene duplication events, conserved protein motifs, and gene expression patterns of 30 *PLL*s in *Populus trichocarpa* (*PtPL1*s). We performed an *in silico* gene expression profiling and quantitative real-time PCR analysis and found that most of the *PtPL1* genes from subgroups Ia and Ib were highly expressed in xylem. *PtPL1-18* from subgroup Ia was preferentially expressed in developing primary xylem and in xylem cells that were developing secondary walls. Overexpression of *PtPL1-18* in poplar reduced plant growth and xylem development. Reduced secondary cell wall thickening and irregular xylem cells were observed in the transgenic trees, probably due to their lower pectin content. Although pectin is not a major component of plant secondary cell walls, our results are consistent with the *PtPL1* genes performing important functions during wood formation.

## Introduction

*Populus* is a genus of trees among the most important boreal broadleaf trees, for its plentiful and renewable supplies of wood in pulping, construction, and energy industries. In addition, it has been proposed to be a sustainable energy source for biofuel production (Carroll and Somerville, 2009). Formation of wood is a sequential developmental process, including vascular cambial division, differentiation of secondary xylem mother cells, cell expansion, massive deposition of secondary walls, programmed cell death, and formation of heartwood (Plomion et al., 2001). Formation of secondary cell wall is essential for wood development and has been comprehensively studied, with focus on cellulose, hemicellulose, and lignin, the three main compositions of wood (Ye and Zhong, 2015). However, the study of relation between pectin and secondary wall was limited. It is known that pectin mainly exists in the primary cell wall and the middle lamella of plants.

Pectin consists of homogalacturonan (HG), rhamnogalacturonan I (RG-I), the substituted galacturonans rhamnogalacturonan II (RG-II), and xylogalacturonan (XGA), which are polysaccharides rich in galacturonic acid (Mohnen, 2008). Pectin is degraded by different pectinases: the acetyl and methoxyl residues of polygalacturonic acids are detached by pectin esterases: pectin acetylesterases (PAEs) and pectin methylesterases (PMEs), respectively; polygalacturonases (PGs) hydrolyze glycosidic bonds between galacturonic acid residues to degrade polygalacturonan; and pectate lyase (PLs) depolymerize pectin polymer by β elimination, bring unsaturated oligosaccharides as a result (Yadav et al., 2009). Plant pectate lyases (PELs, EC 4.2.2.2) belong to polysaccharide lyase family 1 (PL1)^1^. The classification of PELs is based on their activity in cleavage of the α-(1,4)-glycosidic bond between the galacturonic acid units of HG (Marin-Rodriguez et al., 2002; Yadav et al., 2009).

Polysaccharide lyase family 1 enzymes have been extensively characterized in study of plant disease caused by pathogenic microorganisms, such as *Erwinia chrysanthemi*, an extracellular causal agent of soft rot disease in many plant species (Barras et al., 1994). *PL1* genes are expressed in a variety of plant tissues, including pistils (Wu et al., 1996; Kulikauskas and McCormick, 1997), tracheary elements (Domingo et al., 1998; Milioni et al., 2001), latex (Pilatzke-Wunderlich and Nessler, 2001), fibers (Wang H. et al., 2010) and ripening fruits (Marin-Rodriguez et al., 2003).

Genome-wide expression analysis in *Arabidopsis* have suggested PL1s’ functions in growth programmed for cell separation. All *AtPL1* genes are expressed in flowers, several of them are highly expressed in pollen (Palusa et al., 2007; Sun and van Nocker, 2010). In *Populus*, the vascular cambium (VC) and adjacentradial expansion (RE) zone are the sites of highest expression of genes encoding wall-modifying enzymes, including expansins, XETs, cellulases, PMEs, PGs, and pectate lyases (PL1s) (Mellerowicz and Sundberg, 2008). *PtxtPL1-27* is specifically expressed in differentiating xylem at the onset of secondary wall formation. Overexpression of this gene in poplar can substantially modify the extractability of cell wall polymers from xylem tissues and enhance saccharification (Biswal et al., 2014).

However, our knowledge of *PL1* genes in *Populus* and their functions during wood formation is still very limited. In this study, we performed a genome-wide characterization of *PL1* gene family in *P. trichocarpa*, and presented here comprehensive analysis of their phylogeny, gene structure, chromosomal location, gene duplication, conserved motifs, and expression patterns, in aim to identify novel *PL1* candidate genes involved in wood formation. One *Populus PL1* candidate gene, *PtPL1-18*, was further studied by overexpression analysis in hybrid poplar. We found that the *PtPL1-18ox* transgenic plants showed reduced plant height, as well as reduced pectin abundance in xylem tissues compared to control plant. The thickness of cell walls of xylem fiber cells was also reduced, suggesting that this gene was participating in pectin biogenesis for maintaining proper cell wall structure and xylem function in *Populus*.

## Materials and Methods

### Plant Material and Growth Condition

*Populus trichocarpa* Nisqually-1 and 717 hybrid poplar (*P. tremula* × *P. alba*, clone717-1B4) (Leplé et al., 1992) were used for gene cloning and transformation, respectively. Poplar plants were multiplied by tissue culture and subsequently grown in a controlled environmental growth room at a constant 25°C day/night temperature, 16 h photoperiod, light intensity of 100 μmol s^-1^ m^-2^, and relative humidity level of 60%.

### *Pectate Lyase* Gene Family in *P. trichocarpa*

Protein sequences of the 26 known *AtPLL* (*Pectate Lyase-Like*) genes were downloaded from the *Arabidopsis* database (TAIR^2^). A BLASTP search was performed using the protein sequences from Pec_Lyase_C domain of the AtPLL proteins as the query to identify their homologs in poplar from JGI^3^ and Phytozome^4^ (Goodstein et al., 2012). These two websites contain all three versions—v1.1, v2.2 and v3.0—of *P. trichocarpa* genome. Genomic sequences and open reading frames (ORFs) of *PtPL1s* were obtained from Phytozome V11.0 and checked by softberry-FGENESH on line^5^ (Solovyev et al., 2006). The candidate *PtPL1* genes were verified for the presence of a Pec_Lyase_C domain by online analysis program of SMART (Simple Modular Architecture Research Tool^6^) (Letunic et al., 2015). SMART and SignalP 4.0 server^7^ (Petersen et al., 2011) were used to identify the potential signal peptides in PtPL1s. TargetP 1.1 server^8^ (Emanuelsson et al., 2000) was used to predict subcellular localization. The theoretical isoelectric point (pI) and molecular weight (MW) of PtPL1s were calculated using the Compute pI/Mw tool on the ExPASy server^9^ (Gasteiger et al., 2005). Gene structures were displayed with R program. A neighbor joining (NJ) (Saitou and Nei, 1987) tree of *PtPL1*s was generated by MEGA5.1 software (Tamura et al., 2011). Bootstrap analysis was performed with 1000 iterations.

### Multiple Sequence Alignment of PtPL1 Protein Sequences and Motif Analysis

Protein sequences of PtPL1s were imported into BioEdit v7.2.5 (Hall, 1999) and aligned with Clustalx program (Thompson et al., 1997). GENEDOC program (Nicholas et al., 1997) was used to generate the output file. MEME online tool^10^ (Bailey et al., 2006) was used for the conserved motif analysis, with the following parameters: number of repetitions, any; maximum number of motifs, 10; optimum motif width, between 6 and 50; and *e*-value cut off at 1.0 e^-10^.

### Gene Duplication Analysis and Estimation of *K*a/*K*s Ratios

The chromosomal position of all *PtPL1* genes was retrieved from the Phytozome website. The gene ID information of *PtPL1* genes was imported into PopGenIE (The *Populus* Genome Integrative Explorer^11^) (Sjodin et al., 2009) to illustrate their relative position in the chromosomal diagram. Paralogous gene pairs based on phylogenic analysis and chromosomal positional information were selected for the analysis of gene duplication type. The segmental duplicated *PtPL1* genes were identified based on previous annotation (Tuskan et al., 2006). Tandem gene duplications were identified according to the criteria that duplication of five or fewer gene loci occurred within 100 kb distance (Finn et al., 2006; Hu et al., 2010). While the two genes in every pair located on different duplication blocks, they should be the products of retrotransposition (Cao and Li, 2015). To estimate the evolutionary time of duplicated genes, *K*a/*K*s_Calculator software was used to get the *K*a and *K*s values of paralogous gene pairs (Wang D. et al., 2010). *K*s values were converted into duplication time in millions of years (MYA) based on a rate of one substitution per synonymous site per year. The time of duplication events (*T*) was calculated as *T* = *K*s/2λ × 10^-6^ MYA. λ, the clock-like rate, was set as 9.0 × 10^-9^ for *Populus* (Lynch and Conery, 2000).

### *In Silico* Gene Expression Analysis

The expression data of various *PtPL1* genes from different tissues (mature leaves; young leaves; roots; dark-grown seedlings; dark-grown seedlings, etiolated, exposed to light for 3 h; continuous light grown seedlings; female catkins; male catkins and xylem) were obtained from the Poplar eFP browser^12^ (Wilkins et al., 2009). The probe sets of the *PtPL1* genes were obtained by the probe match tool from NetAffx^TM^ Analysis center^13^. A heat map was generated based on the gene expression data, using the MultiExperiment Viewer Program (MeV 4.7.3) (Howe et al., 2010).

### RNA Extraction and qRT-PCR Analysis

Tissue samples were collected in August of 2014 from a 3-year-old 717 poplar plant in the campus of Huazhong Agricultural University, Wuhan, China. Fifteen tissues were analyzed: apex (A), dormant apex (DA), dormant lateral bud (DLB), the 2nd, 3rd, 4th, 5th, and 9th internode of the main stem (IN2, IN3, IN4, IN5, and IN9, respectively), young leaves (YL), mature leaves (ML), petiole (P), primary root (PR), secondary root (SR), xylem and cambium (XC), phloem and cambium (PC). All samples were collected in triplicates and immediately frozen in liquid nitrogen. Total RNA was extracted using CTAB method (Li et al., 2008). The quality and concentration of each RNA sample was determined using a NanoDrop 2000 spectrophotometer (Thermo Scientific). RNA samples that met the criterion (A_260_/A_280_ ratio of 1.8–2.1, A_260_/A_230_ ratio ≥ 2.0) were stored at -80°C for further use. The cDNA was synthesized from 0.5 μg RNA using the PrimeScript^TM^ RT Kit (TaKaRa, Dalian). qRT-PCR was performed in duplicates using 2×SYBR qPCR Mix without ROX (ZOMANBIO, Beijing) on a 7500 Fast Real-Time PCR System (Applied Biosystems). *PtACTIN* (*Potri.019G010400*) was used as reference gene. The sequences of qRT primers for *PtPL1* genes were listed in **Supplementary Table S1**. ΔΔCt method was used to calculate gene relative expression (Pfaffl, 2001). All reactions were repeated with three biological replicates.

### Plasmid Construction and Generation of Transgenic Plants

For the construction of overexpression lines, the CDS region of *PtPL1-18* was amplified by PCR and cloned into Gateway^TM^ entry vector pDONR201. The vector with the correct sequences was subsequently recombined into destination vector pK2GW7 with promoter CaMV35S, to generate destination vector ready for *Agrobacterium* and *Populus* transformation. For promoter activity analysis, the 2.0 kb upstream promoter region of *PtPL1-18* was amplified by PCR and cloned into pDONR201. The verified entry vector was recombined into destination vector pKGWFS7 (Karimi et al., 2002). *Agrobacterium tumefaciens* strain C58 was used for poplar transformation (Leplé et al., 1992). Positive transgenic lines were selected by Kanamycin resistance, then verified by PCR with specific primers (**Supplementary Table S1**) for each fragments. Overexpression lines were further characterized by semi-quantitative RT-PCR with 30 cycles used specific primers in **Supplementary Table S1** to test the transcript level of *PtPL1-18*. To estimate the increase of transcript accumulation level in three selected over-expressing plants, the 12th internodes of 4-month-old *PtPL1-18* overexpression plants and 717 plants were collected to conduct qRT-PCR analysis with above methods. *PtACTIN* (*Potri.019G010400*) was used as reference gene. The sequences of qRT-PCR primers were listed in **Supplementary Table S1**.

### Anatomical and Histochemical Analysis of Transgenic Plants

Hybrid poplar 717 and three independent *PtPL1-18* overexpression lines were propagated by tissue culture and transferred to soil. Plant height and stem diameter were measured after 4 months of growth, from six randomly selected individual plants per line. Cross sections were made from the 12th internodes and the corresponding petioles using vibratome (Leica VT 1200S, Germany). The sections were stained with 5% (w/v) phloroglucinol in 12% (v/v) HCl for indication of lignification, and observed under bright field microscope (Olympus BX63, Japan) for digital imaging.

For GUS assay, internode 4, 7, and 12 of the main stem and their corresponding petioles were collected from transgenic plants carrying *PtPL1-18pro:GUS*, washed twice with 50 mM sodium phosphate buffer (pH 7.0), and incubated in X-gluc reaction solution (1 mM X-gluc, 50 mM sodium phosphate, pH 7.0, 0.1% Triton X-100, 1 mM potassium ferricyanide and 1 mM potassium ferrocyanide) at 37°C overnight. Then 40 μm thick cross sections were prepared using vibratome (Leica VT 1200S, Germany), cleared with 75% ethanol and observed under bright-field microscope (Olympus BX63, Japan).

### Cell Wall Composition Analysis of *PtPL1-18* Overexpression Lines

The 12th internodes of 4-month-old *PtPL1-18* overexpression plants and 717 plants were collected. The material for cell wall composition analysis was inactivated at 105°C for 30 min, dried to constant weight at 50°C, grounded into powder and filtered through a 40 mesh screen. A cell wall polymer fractionation procedure described previously (Wu et al., 2013) was used. The soluble sugar, lipids and starch of the biomass samples were consecutively removed by potassium phosphate buffer (pH 7.0), chloroform-methanol (1:1, v/v) and DMSO-water (9:1, v/v). The crude cell wall material was suspended in 0.5% (w/v) ammonium oxalate and heated for 1 h in a boiling water bath, and the supernatants were combined as total pectin. The remaining pellet was suspended in 4M KOH containing 1.0 mg mL^-1^ sodium borohydride for 1 h at 25°C, and the combined supernatant was neutralized, dialyzed and lyophilized as hemicelluloses. The remaining pellet was extracted with H_2_SO_4_ (67%, v/v) for 1 h at 25°C and the supernatants were collected for determination of free hexoses and pentoses as total cellulose and non-KOH-extractable hemicelluloses. The total hemicelluloses is the sum of KOH- and non-KOH-extractable hemicelluloses. UV–VIS Spectrometer (V-1100D, Shanghai MAPADA Instruments Co., Ltd., Shanghai, China) was used for the absorbance reading according to Xu et al. (2012). The total lignin is the sum of acid-insoluble and acid -soluble lignin. The acid-insoluble lignin was calculated gravimetrically as acid-insoluble residue after correction for ash, and the acid-soluble lignin was measured by UV spectroscopy. All experiments were carried out in triplicate.

### Immunolocalization of Pectin Epitopes in Stem of *PtPL1-18* Overexpression Lines

The 7th internodes from 2-month-old poplar propagated by tissue culture were fixed for 2.5 h in 4% (w/v) paraformaldehyde. Tissues were dehydrated through an ethanol series (30, 40, 50, 60, 75, 80, 95, and 100% [v/v]) for 40 min and embedded in paraffin. Samples were sectioned with 8 μm thickness using a Leica RM2265 rotary microtome. After the removal of paraffin, sections were blocked with 3% (w/v) non-fat milk in 10–15 μL 10 mM potassium phosphate buffered saline (KPBS, pH = 7.1) for 1 h. The slides were pretreated with poly-lysine. All of the following steps were conducted in a petri dish chamber with soaked tissue paper to keep certain humidity level. Sections were incubated with primary antibodies (10–15 mL of each sample) for 2 h at room temperature. CCRC-M35 (Pattathil et al., 2010) and JIM5 (Clausen et al., 2003) monoclonal antibodies were used in this analysis, with a dilution factor of 5- and 10-fold, respectively. Sections were subsequently washed three times with 10 mM KPBS, each step for at least 5 min. The sections were incubated with anti-mouse Alexa fluor 488 [IgG (H + L), Invitrogen, A11001] for CCRC-M35 and anti-rat Alexa fluor 488 [IgG (H + L), Invitrogen, A-11006] for JIM5, respectively. The two secondary antibodies were linked to Fluorescein-isothiocyanate (FITC), and a 100-fold dilution in 10 mM KPBS was used. Sections were washed at least three times again with 10 mM KPBS and finally with water before visualized under Olympus BX-61 fluorescent microscope (Avci et al., 2012). All experiments were carried out in triplicate.

### Statistical Analysis

The experimental data was subjected to Analysis of Variance (ANOVA) using SAS version 8.0 (SAS Institute Inc., Cary, NC, United States). ^∗∗^ or ^∗^ indicated significant difference between means at *p* < 0.01 or *p* < 0.05, respectively, by Student’s *t*-test.

## Results

### Characterization of *PtPL1* Genes in *P. trichocarpa*

The Pec_Lyase_C domain of the AtPLL proteins was used as the query to identify their homologs in poplar from JGI and Phytozome database by BLASTP search tool. In total, there were 30 full-length *PtPL1* genes in the *P. trichocarpa* genome. Twenty-eight of them have been previously identified in the first version of *Populus* genome, named as *PtPL1-1* to *PtPL1-28* (Geisler-Lee et al., 2006); and one member was identified in the *Populus* assembly v2.2, named as *PtPL1-29* (Biswal et al., 2014). In this study, a novel *PtPL1* gene (Potri.012G091300) was identified in the *Populus* assembly v3.0, named as *PtPL1-30* (**Supplementary Table S2**).

**FIGURE 1:**
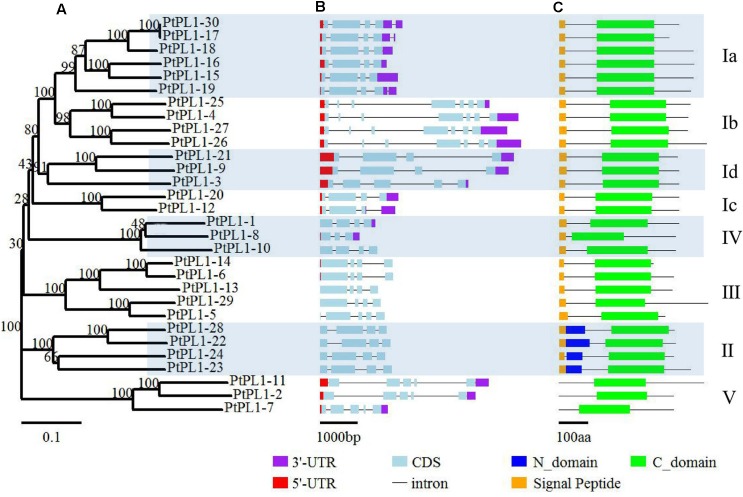
Phylogenetic relationships, gene structure and conserved domains of *PtPL1* gene family. The 30 full-length PtPL1 protein sequences were aligned by ClustalX2, an un-rooted Neighbor-Joining (NJ) tree **(A)** was developed by the MEGA5.0.5 program. Bootstrap analysis was performed with 1000 iterations, numbers on the nodes indicated clade credibility values. *PtPL1-1* to *PtPL1-28* were previously identified by Geisler-Lee et al. (2006) and *PtPL1-29* was identified by Biswal et al. (2014). *PtPL1-30* is a novel gene in this study. The subgroups followed Biswal et al. (2014) Gene structures of *PtPL1* family genes were shown in color scheme (3′-UTR in purple, 5′-UTR in red, exon in blue) **(B)**. Conserved domains in PtPL1 proteins were predicted by SMART website (http://smart.embl-heidelberg.de/) **(C)**. Green boxes: Pec_lyase_C domain (C-domain); Dark blue boxes: Pec_lyase_N domain (N-domain); Yellow boxes: Signal peptide.

The *PtPL1* gene family was classified into five groups—I, II, III, IV, and V—based on phylogenetic analysis (**Figure 1**) (Biswal et al., 2014). Group I had the most members and could be further classified into four subgroups—Ia, Ib, Ic, and Id. The newly identified *PtPL1-30* gene belonged to subgroup Ia. Gene structures of all *PtPL1* genes were predicted to confirm the conservation within each group/subgroup (**Figure 1**). The coding regions of *PtPL1* genes were interrupted by two to six introns: subgroup Ib possessed six introns, while other group/subgroup had an average of three to four introns. Each group/subgroup shared similar gene structure in terms of the number and the length of exons and introns.

The length of PtPL1 proteins were ranging from 266 to 496 amino acid residues. The theoretical pI and MW of PtPL1s were predicted using the Compute pI/Mw tool on the ExPASy server^14^, showing a range of 5.58–9.67 and 29.5–53.7 kDa, respectively (**Supplementary Table S3**). All the PtPL1 proteins were verified for the presence of a Pec_Lyase_C domain by online analysis program of SMART^15^. Another conserved domain, Pec_lyase_N, was predicted in the four members of Group II in addition to the Pec_Lyase_C domain. Most members of PtPL1 family were predicted to contain an N-terminal signal peptide (cutoff > 0.45), except for the three members from Group V (**Figure 1**). The prediction of subcellular localization showed that members in subgroup I, II, III, and IV were predicted as signal peptide secretion pathway. PtPL1-2 was predicted to have a chloroplast transit peptide while no particular sub-cellular localization could be predicted for PtPL1-7 and PtPL1-11, which were members in Group V (**Supplementary Table S4**).

### Chromosomal Location and Duplication Analysis of *PtPL1* Genes

**FIGURE 2:**
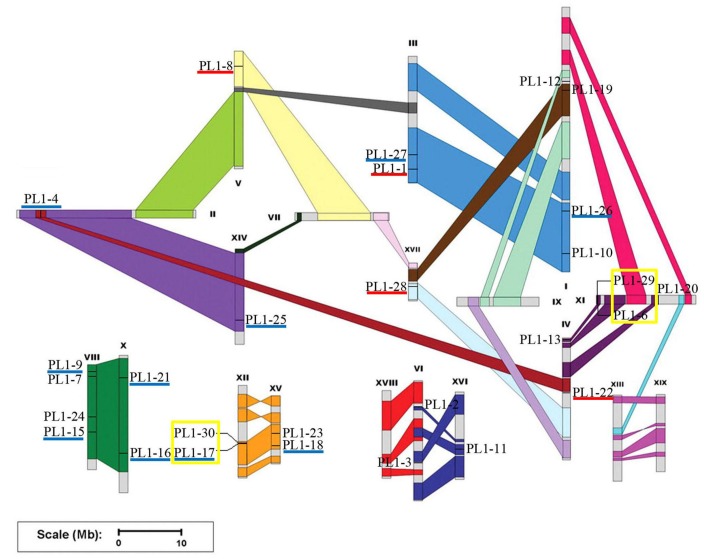
Chromosomal location of the *PtPL1* genes on *Populus trichocarpa* genome. The annotation information of *PtPL1* genes from Phytozome V11.0 was imported into PopGenIE (http://popgenie.org/). Twenty-eight *PtPL1* genes are randomly distributed on 14 out of 19 linkage groups; the rest two genes are assigned to unassembled scaffold (**Supplementary Table S2**). The schematic representation of *Populus* chromosome organization with whole-genome duplication events was obtained from Tuskan et al. (2006). Segmental duplicated homologous regions were shown with the same color. Five pairs of segmental duplicated genes were underlined in dark blue. Tandemly duplicated genes were displayed with yellow boxes. Paralog genes arising from retro-transposition were underlined in red.

Chromosomal location of the *PtPL1* genes was illustrated in **Figure 2** based on the gene information from *P. trichocarpa* (v3.0) in Phytozome^16^. 28 *PtPL1* genes were randomly distributed on 14 out of 19 linkage groups (**Figure 2**); the rest two genes, *PtPL1-5* (Potri.T040400) and *PtPL1-14* (Potri.T040300), were assigned to individual scaffolds.

Based on phylogenetic analysis and chromosomal location of the *PtPL1* genes, 13 potential paralogous gene pairs (**Table 1**) were selected for duplication analysis. It is most likely that these paralogous gene pairs were created via genome duplication events (Tuskan et al., 2006). To determine the origin of these 13 paralogous pairs, i.e., the type and the exact time of the duplication events, we calculated the *K*a and *K*s values for each of the paralog gene pairs (**Table 1**). The result showed that five paralogous pairs, *PtPL1-17*/*PtPL1-18*, *PtPL1-16*/*PtPL1-15*, *PtPL1-27*/*PtPL1-26*, *PtPL1-25*/*PtPL1-4*, and *PtPL1-21*/*PtPL1-9* were segmental duplications. These paralog pairs were shown in the same color block on different chromosomal locations in **Figure 2**. Synonymous substitution values (*K*s) suggested that the segmental duplication occurred ca. 13.89–19.95 million years ago (**Table 1**). These periods coincide with the time of *P. trichocarpa* genome recent duplication events (Tuskan et al., 2006). On the other hand, *PtPL1-30*/*PtPL1-17* and *PtPL1-6/PtPL1-29*, which were likely to be a result of tandem gene duplication (Finn et al., 2006; Hu et al., 2010), additional that, *PtPL1-6* and *PtPL1-29* both had a paralogous gene in the phylogenetic tree, *PtPL1-14* and *PtPL1-5*, respectively. Interestingly, *PtPL1-14* and *PtPL1-5* were located on scaffold_38 based on *Populus* genome assembly V3.0, but they were located on chromosome 11 according to *Populus* genome assembly V2.2. Despite this, we also suspect that *PtPL1-6*, *PtPL1-29*, *PtPL1-14* and *PtPL1-5* were tandem genes on chromosome 11. The estimated time of duplication of *PtPL1-14*/*PtPL1-6* and *PtPL1-29*/*PtPL1-5* was 1.43 and 1.51 mya, respectively; and the estimated age of *PtPL1-30*/*PtPL1-17* was 0.40 mya. Of the other two pairs, *PtPL1-1*/*PtPL1-8* and *PtPL1-28*/*PtPL1-22*, the two genes in every pair were located on different duplication blocks. They were likely the products of retro-transposition (Chen and Cao, 2014; Cao and Li, 2015), and the estimated age was 21.81 and 19.80 mya, respectively. While duplications of *PtPL1-20*/*PtPL1-12* and *PtPL1-24*/*PtPL1-23* occurred 109.45 and 113.34 mya, respectively, nearly within or following the time when *Populus* and *Arabidopsis* lineages diverged (Tuskan et al., 2006). It was suggested that *PtPL1-20*/*PtPL1-12* and *PtPL1-24*/*PtPL1-23* were from more ancient segmental duplication. At the same time, we calculated the *K*a, *K*s, *K*a/*K*s of *PtPL1* genes (**Table 1**). The estimated *K*a/*K*s values of most gene pairs were less than 1.0 except that the *K*a/*K*s value of *PL1-20*/*PL1-12* was 1.4.

**Table 1:** Inference of duplication time of *PtPL1* paralogous pairs.

Paralogous pairs	*K*a	*K*s	*K*a/*K*s	Duplication types	MYA
*PL1-30*/*PL1-17*	0.0011	0.0073	0.1472	Tandem	0.40
*PL1-17*/*PL1-18*	0.0234	0.357	0.0656	Segmental	19.62
*PL1-16*/*PL1-15*	0.0384	0.344	0.1117	Segmental	18.90
*PL1-27*/*PL1-26*	0.035	0.2528	0.1383	Segmental	13.89
*PL1-25*/*PL1-4*	0.0414	0.2623	0.1579	Segmental	14.41
*PL1-21*/*PL1-9*	0.0628	0.363	0.1731	Segmental	19.95
*PL1-20*/*PL1-12*	2.7887	1.9919	1.4000	Segmental	109.45
*PL1-1*/*PL1-8*	0.0495	0.3969	0.1248	Retrotransposition	21.81
*PL1-14*/*PL1-6*	0.0067	0.0261	0.2547	Tandem	1.43
*PL1-29*/*PL1-5*	0.0046	0.0275	0.1678	Tandem	1.51
*PL1-28*/*PL1-22*	0.0752	0.3604	0.2086	Retrotransposition	19.80
*PL1-24*/*PL1-23*	0.2143	2.0628	0.1039	Segmental	113.34
*PL1-11*/*PL1-2*	0.0469	0.2795	0.1677	Retrotransposition	15.36


### Multiple Sequence Alignment and Motif Compositions of PtPL1 Proteins

Multiple sequence alignment of PtPL1 proteins with Pec_lyase_C domain was conducted using ClustalX software and output with GENEDOC program (**Supplementary Figure S1**). All the 30 PtPL1 proteins contained a Pec_lyase_C domain, including four residues for Ca^+2^ binding, five residues for substrate binding, one residue for catalysis and one residue for disulfide bond (Herron et al., 2003). The corresponding residues were shown in brown, blue, green, and pink, respectively. Most of these residues were conserved among the 30 PtPL1 proteins, except for the members from group V. Group V had an arginine (R) to replace the histidine (H) at the second residue for substrate binding; and there was no cysteine (C) at the residue for disulfide bond in this group.

**FIGURE 3:**
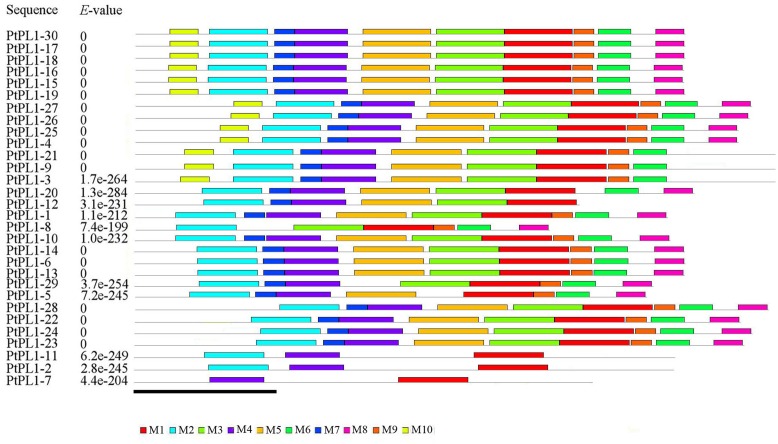
Conserved protein motif analysis of PtPL1s by MEME. Conserved motifs in the PtPL1 proteins were elucidated by MEME website (http://meme-suite.org/tools/meme). Up to 10 motifs were shown by different colors. The height of the motif “block” is proportional to *p*-value, truncated at the height for a motif with a *p*-value of 1*e*^-10^. MEME Parameters: number of repetitions, any; maximum number of motifs, 10; optimum motif width, between 6 and 50. Bar = 100 amino acids.

To further reveal the specific regions of different PtPL1 proteins, motif analysis was performed using MEME online tool^17^ (**Figure 3**). In general, PtPL1 proteins from the same group shared similar motifs. The length of motifs ranged from 6 to 50 amino acid residues; and the number of motifs varied between 2 and 10 in each PtPL1 protein. Motif 1, 2, 4, 7 were present in most of the PtPL1 proteins. Motif 7 (blue), with the first two residues for Ca^+2^ binding and the first two residues for substrate binding, was absent in PtPL1-10 and the whole group V. Motif 2 (cyan), with the residue for disulfide bond, the last two residue for Ca^+2^ binding and the third residue for substrate binding, was absent in PtPL1-7. Motif 1 (red), with the residue for catalysis and the 4th and 5th residue for substrate binding, was present in all PtPL1 proteins.

### Expression Analysis of the *PtPL1* Genes in Different Tissues of Poplar

**FIGURE 4:**
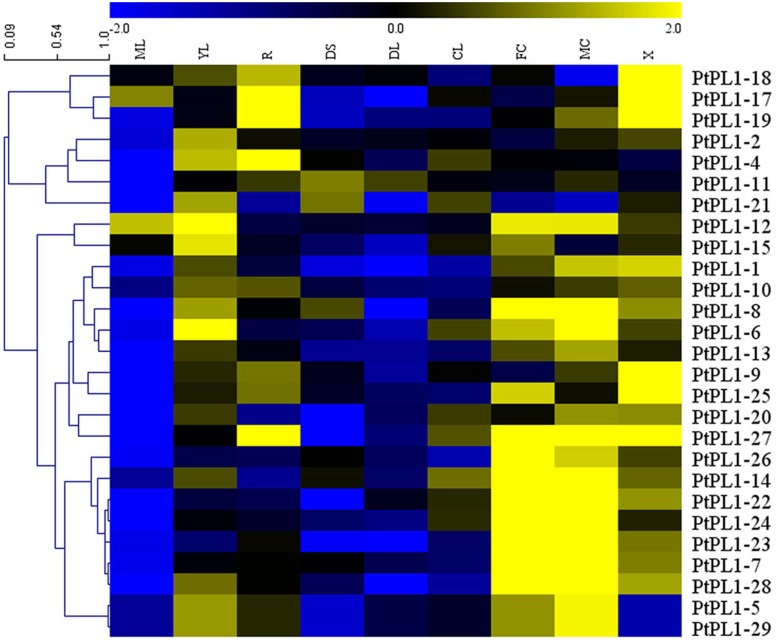
Expression profiles of the *PtPL1* genes. A heat map was generated with microarray data of 27 *PtPL1* genes in nine different tissues from Poplar eFP Browser (http://bbc.botany.utoronto.ca/efppop/) (Wilkins et al., 2009). The transcript levels for the *PtPL1* genes were clustered using hierarchical clustering based on Pearson correlation. Color scale on top represented LN2 from –2.0 to 2.0. Blue indicated low expressed genes, while yellow indicated highly expressed genes. Abbreviation for tissues are as following: ML, mature leaf; YL, young leaf; R, root; DS, dark-grown seedlings; DL, dark-grown seedlings, etiolated, then exposed to light for 3 h; CL, continuous light grown seedling; FC, female catkins; MC, male catkins; X, xylem.

**FIGURE 5:**
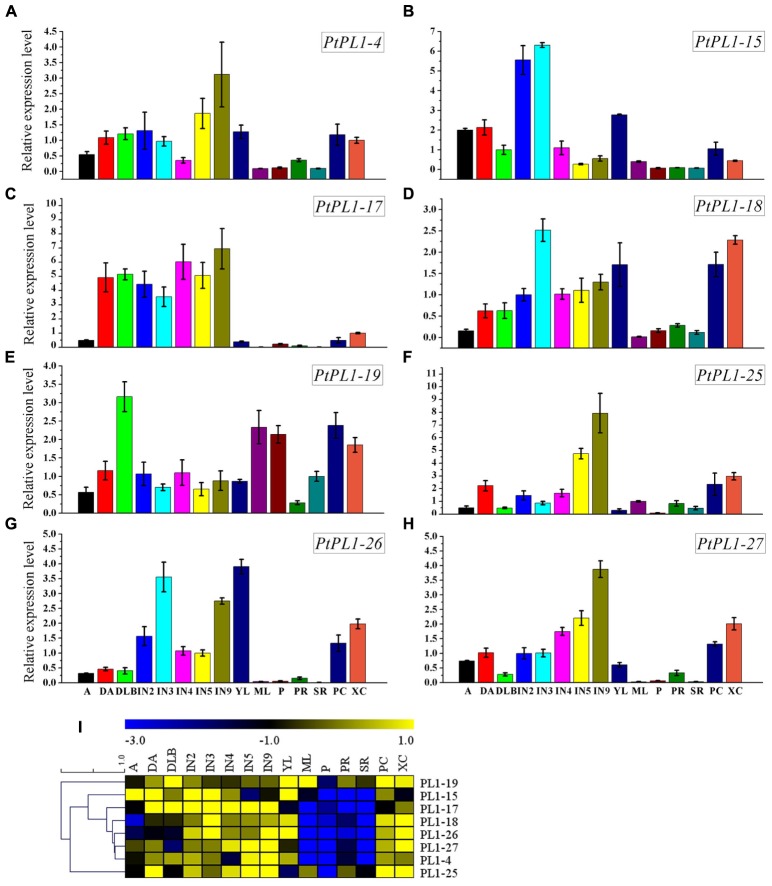
Relative transcript levels of selected *PtPL1* genes. **(A–H)** Relative transcript levels of eight selected *PtPL1* genes by real-time quantitative RT-PCR. *PtACTIN* (*Potri.019G010400*) was used as reference gene, using the ΔΔCt method. Standard deviation was calculated from three biological replicates. **(I)** A heat map for comparison of relative expression of eight selected *PtPL1* genes in 15 tissues tested. Color scale represented LN2 from –3.0 to 1.0. Tissues tested: apex (A), dormant apex (DA), dormant lateral bud (DLB), the 2nd internode stem (IN2), the 3rd internode stem (IN3), the 4th internode stem (IN4), the 5th internode stem (IN5), the 9th internode stem (IN9), young leaves (YL), mature leaves (ML), petiole (P), primary root (PR), secondary root (SR), phloem and cambium (PC), xylem and cambium (XC).

To investigate the expression pattern of all *PtPL1* genes in different tissues, a set of Affymetrix microarray data from the Poplar eFP browser (Wilkins et al., 2009) were downloaded and analyzed. The probe sets matched to each *PtPL1* gene were listed in **Supplementary Table S5**. Expression data of 27 *PtPL1* genes were obtained, except for *PtPL1-3*, *16*, and *30*. A heatmap based on the expression data was generated using MeV 4.7.3 (**Figure 4**). The heatmap showed that most of *PtPL1* genes were highly expressed in female catkins (FC), male catkins (MC), xylem (X), root (R), or young leaf (YL). Of particular interest to us is that six *PtPL1* genes—*PtPL1-9*, *17*, *18*, *19*, *25*, and *27*—had the highest expression level in xylem and root (**Figure 4**). Five of them belonged to subgroup Ia and Ib, with *PtPL1-9* from subgroup Ic as the only exception. In order to find candidate *PtPL1* genes involved in the development of vascular tissues, expression of members from subgroup Ia and Ib were further investigated by qRT-PCR (**Figure 5**). A set of samples related to vascular development were tested. Expression data of eight *PtPL1* genes were obtained, except for that of *PtPL1-16* and *PtPL1-30*. Most of the selected genes were preferentially expressed in stem (**Figures 5A–H**). A heatmap was generated and co-expression analysis of the eight *PtPL1* genes was performed using MeV 4.7.3 (**Figure 5I**). One *PtPL1* gene from subgroup Ia, *PtPL1-18*, displayed a high correlation with *Ptxt1-27* (**Figure 5I**), which is from Ib and specifically expressed in differentiating xylem (Biswal et al., 2014). Based on the above-mentioned phylogenetic analysis, expression and co-expression analysis, *PtPL1-18* was chosen as the candidate gene from subgroup Ia for the following functional studies during vascular tissue development.

### Histochemical Analysis of Petioles and Stems in the *PtPL1-18pro:GUS* Lines

**FIGURE 6:**
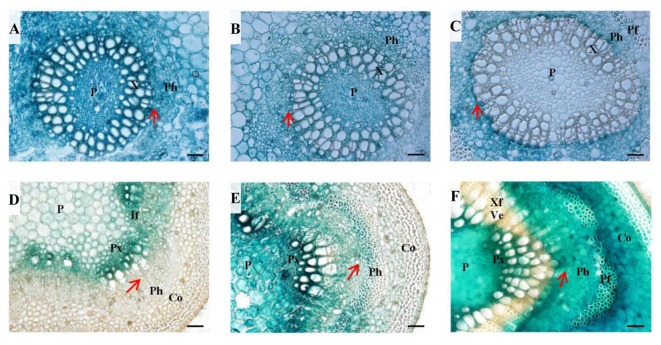
Histochemical analysis in *PtPL-18pro:GUS* transgenic poplar leaf petioles and stems. Four-month-old plants grown in the greenhouse were used for transverse section of leaf petioles **(A–C)** and stems **(D–F)** followed by GUS staining. **(A)** LPI 4 leaf petiole, **(B)** LPI 7 leaf petiole, **(C)** LPI 12 leaf petiole, **(D)** internodes 4, **(E)** internode 7, **(F)** internode 12. P, pith; X, xylem; Ph, phloem; Pf, phloem fiber; Px, primary xylem; If, interfascicular; Co, cortex; Ve, vessel element; Xf, xylem fiber. The red arrows represent vascular cambium. Bar = 50 μm.

To have a detailed view of the expression of *PtPL1-18* in vascular development, *PtPL-18pro:GUS* transgenic lines of poplar 717 were prepared for histochemical analysis. Transverse sectioning of both petiole and stem at different developmental stages was performed in 4-month-old plants of three representative transgenic lines. In the 4th leaf petioles, GUS staining was clearly detected in all cell types in vascular bundle (**Figure 6A**). The staining was getting weaker especially in xylem upon the 7th leaf petioles. In the 12th leaf petioles, GUS staining could not be detected neither in mature xylem, nor in pith, but remained in phloem, pro-cambium and developing xylem (**Figures 6B,C**). In parallel, the GUS expression patterns in corresponding internodes were studied. In the 4th stem, the GUS activity was preferential in primary xylem and interfascicular region (**Figure 6D**). The GUS staining in internode 7 was stronger and broader than the 4th internode; and it was detected in all cell types of the vascular bundle, including the central pith. The GUS staining was strongest in primary xylem. Weak staining was also detected in cortex (**Figure 6E**). In internode 12, the GUS staining covered the cortex, phloem and pith areas, normally in parenchyma cells, as well as in phloem fiber cells, primary xylem cells and developing xylem cells, but not in mature xylem, in which secondary cell wall has formed in xylem fiber cells and vessel cells (**Figure 6F**).

### Effects of *PtPL1-18* Overexpression on Secondary Cell Wall Formation and Vascular Development

**FIGURE 7:**
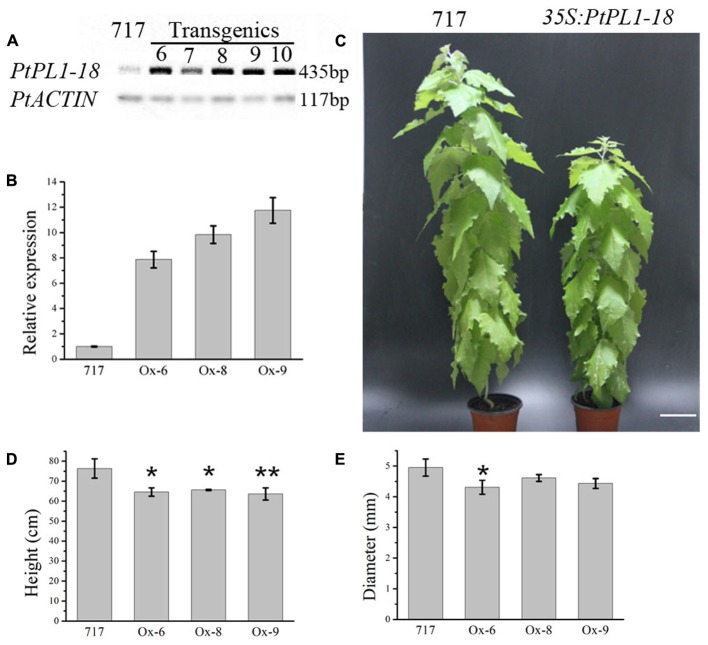
Effects of *PtPL1-18* overexpression on *Populus* growth. **(A)** Characterization of *PtPL1-18* expression level in *35S:PtrPL1-18* transgenic lines. Poplar 717 was served as control. *PtACTIN* was used as the reference gene in RT-PCR. **(B)** *PtPL1-18* relative transcript levels of poplar 717 and 3 selected *PtPL1-18* overexpression lines by real-time quantitative RT-PCR. *PtACTIN* (*Potri.019G010400*) was used as reference gene, using the ΔΔCt method. Standard deviation was calculated from three biological replicates. **(C)** Picture of 4-month-old *PtPL1-18ox* (*35S:PtPL1-18*) transgenic poplar (right) compared to poplar 717 (left). Bar = 10 cm. **(D)** Plant height and **(E)** basal stem diameter of 4-month-old *PtPL1-18ox* transgenic poplar plants. Data for poplar 717 and transgenic plants were calculated from six individual plants (*n* = 6). ^∗^ and ^∗∗^ indicated significant differences at *P* < 0.05 and *P* < 0.01 by Student’s test, respectively.

A full length *PtPL1-18* CDS was cloned into pK2GW7 to obtain overexpression lines. The transcript levels of *PtPL1-18* in *35S:PtPL1-18* overexpression lines were verified by semi-quantitative RT-PCR. Out of 15 transgenic lines, five lines (line 6–10) showed significantly elevated transcript level of *PtPL1-18* (**Figure 7A**). Lines 6, 8, and 9 were used for the following study. Poplar 717 and the three *PtPL1-18* overexpression lines were propagated by tissue culture and transferred to soil. The increase of transcript accumulation level in over-expressing plants was estimated by qRT-PCR. Compared with 717 poplar, *PtPL1-18* transcript levels were 7.9, 9.8, 11.8 times higher in the stem of overexpression lines 6, 8, and 9, respectively (**Figure 7B**). Plant height and stem diameter were measured after 4 months in soil. *PtPL1-18* overexpression lines had reduced plant height compared to poplar 717 (**Figure 7C**). The average reductions on plant height in lines 6, 8, and 9 were 15.43, 14.04, and 16.66%, with *p*-value of <0.05, <0.05, and <0.01, respectively (**Figure 7D**). The diameter of stem of overexpression lines was reduced slightly (**Figure 7E**).

**FIGURE 8:**
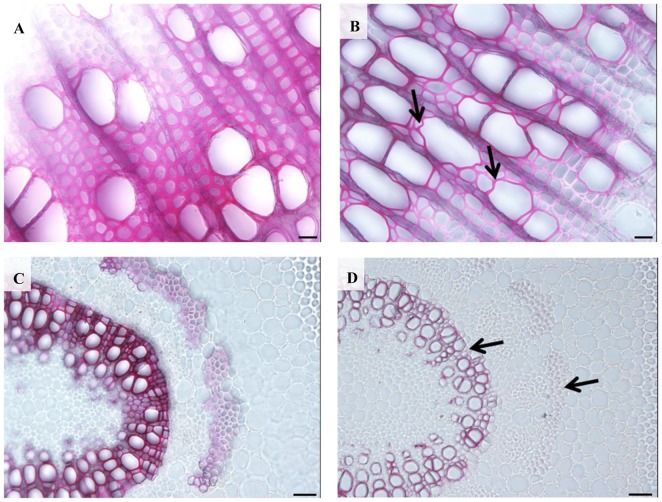
Effects of *PtPL1-18* overexpression on *Populus* stem development. **(A,B)** Stem cross sections from the 12th internodes of poplar 717 **(A)** and *PtPL1-18ox* transgenic plants **(B)** by phloroglucinol–HCl staining. Deformed vessel in *PtPL1-18ox* stem were indicated by black arrows. **(C,D)** Transverse section of the 12th leaf petiole of poplar 717 **(C)** and *PtPL1-18ox* transgenic plants **(D)**. Reduced wall thickness of xylary fibers and phloem fiber cells in *PtPL1-18ox* petioles were indicated by arrowheads. **(A,B)** Bars = 20 μm, **(C,D)** Bars = 50 μm.

When we examined the stem cross-sections from internode 12, an obvious irregular-xylem phenotype (collapsed vessel elements) in *35S:PtPL1-18* transgenic plants was found (**Figure 8B**). Both xylary fiber and vessel cells from the *35S:PtPL1-18* transgenic plants showed much thinner secondary walls than that from control plant (**Figures 8A,B**). We also analyzed the cross-sections of 12th leaf petiole in *35S:PtPL1-18* transgenic plants and control (**Figures 8C,D**). Similarly, the xylary fiber, phloem fiber cells also showed delayed secondary wall thickening in transgenic plants compared to the control, however, there was no difference for the cell size of the vessel elements or fiber cells (**Figures 8C,D**).

**FIGURE 9:**
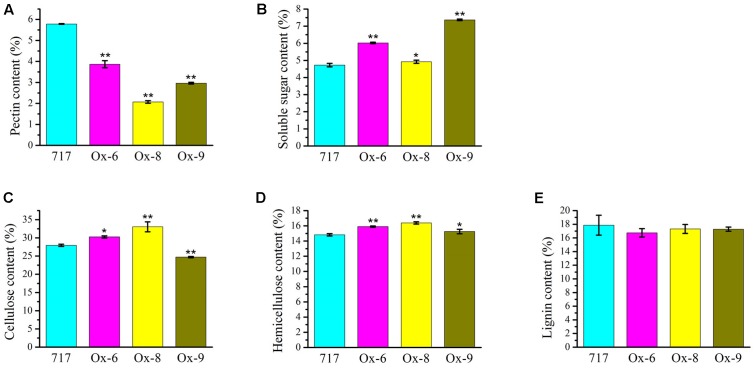
Cell wall compositions in the stems of poplar 717 and *35S:PtPL1-18* transgenic plants. Cell wall composition was determined, using the 12th internodes of 4-month-old plant of *PtPL1-18ox* transgenic lines (Ox-6, Ox-8, and Ox-9), with poplar 717 as control. The content of pectin **(A)**, soluble sugar **(B)**, cellulose **(C)**, hemicellulose **(D)**, and lignin **(E)** were presented as weight percentage of the dry mass (w/w %). Standard deviation was calculated from three biological replicates, ^∗^ and ^∗∗^ indicated significant difference at *P* < 0.05 or *P* < 0.01, respectively (Student’s *t*-test).

Upon the observation on the reduced plant height and thinner secondary cell wall phenotype of the *35S:PtPL1-18* transgenic plants, we further characterized the cell wall composition between overexpression lines and poplar 717 (**Figure 9**). The results revealed that the total pectin content was significantly reduced in the *35S:PtPL1-18* overexpression lines (**Figure 9A**), along with an increase of soluble sugar (**Figure 9B**). The content of cellulose was increased in line Ox-6 and Ox-8, and the average increments were 8.26%, 18.19% compared to poplar 717, with *p*-value of <0.05 and <0.01, respectively; on the contrary, the content of cellulose was decreased in line Ox-9, the average reduction was 11.60%, with *p*-value of <0.01 (**Figure 9C**). The hemicellulose content was increased in all the three lines, and the average increments in line Ox-6, Ox-8, and Ox-9 were 7.30, 10.49, and 3.74% compared to poplar 717, with *p*-value of <0.01, <0.01, and <0.05, respectively (**Figure 9D**). While the lignin content did not significantly vary between three *35S:PtPL1-18* overexpression lines and poplar 717 (**Figure 9E**).

### CCRC-M35 and JIM5 Binding to Cell Walls in Poplar Stems Is Decreased by Overexpression of *PtPL1-18*

**FIGURE 10:**
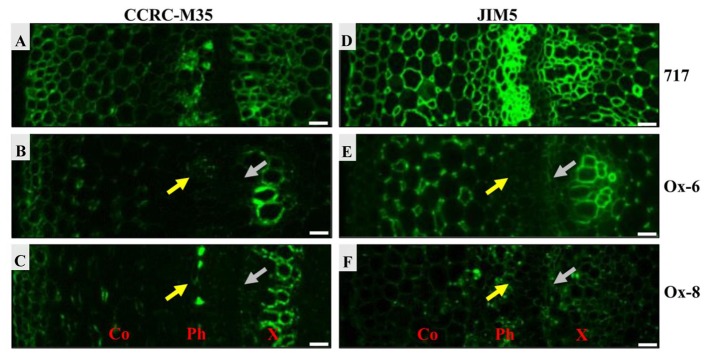
Immunofluorescence detection of pectic HG and RGI epitopes in stems of 717 poplar and two independent *PtPL1-18* overexpression lines. Immunofluorescence detections of RGI in poplar 717 **(A)** and two independent *PtPL1-18* overexpression lines Ox-6 **(B)**, Ox-8 **(C)** stem transverse sections by CCRC-M35. The immunolabel with CCRC-M35 showed RGI was across cell walls and abundant at phloem, primary xylem and developing xylem **(A)**. In Ox-6 **(B)** and Ox-8 **(C)**, CCRC-M35 epitope has been weaker obviously in phloem (yellow arrows) and developing xylem (gray arrows). Immunofluorescence detections of HG in poplar 717 **(D)** and Ox-6 **(E)**, Ox-8 **(F)** by JIM5. JIM5 bound to all cell walls in the stem of poplar 717 **(D)**. And the signal was strong in phloem, primary xylem and developing xylem. In Ox-6 **(E)** and Ox-8 **(F)** transgenic plants, the immunolabel with JIM5 showed HG less in phloem and developing xylem compared to poplar 717. Co, cortex; Ph, phloem; X, xylem; Bars = 20 μm.

Two antibodies, CCRC-M35 and JIM5, were used to investigate the distribution of pectic polysaccharides in stem sections (**Figure 10**). CCRC-M35 binds to the backbone of RG-I and requires at least two unbranched disaccharide repeats for the binding (Pattathil et al., 2010). As shown in **Figure 10**, the RG-I epitope was present throughout the extracellular spaces. The RG-I signal was quite prominent in cortex, phloem, and xylem tissues in the stem sections of poplar 717 (**Figure 10A**), while the signal was much weaker in phloem and developing xylem in *PtPL1-18* Ox-6 and Ox-8 transgenic plants (**Figures 10B,C**), suggesting a reduced pectin contents in those tissues. The difference in HG content, detected by JIM5 antibody was more evident (**Figures 10D–F**). JIM5 recognizes partially methylesterified HG (Clausen et al., 2003). JIM5-HG epitope was detected throughout cell boundaries in the stem sections of poplar 717, particularly in phloem and xylem tissues (**Figure 10D**). The signal was much weaker in phloem and developing xylem in *PtPL1-18* Ox-6 and Ox-8 plants (**Figures 10E,F**), as compared to poplar 717.

## Discussion

### Characterization of 30 *PtPL1* Genes in *Populus*

In prior study, over 1,600 genes encoding carbohydrate-active enzymes (CAZymes) in the *Populus trichocarpa* genome were identified and grouped into families of glycosyltransferases, glycoside hydrolases, carbohydrate esterases, polysaccharide lyases, and expansins. Among them, 28 *PL1* genes were identified and named as *PL1-1* to *28* (Geisler-Lee et al., 2006). In another study, *PL1-29* was identified (Biswal et al., 2014). In this study, *PtPL1-30* (*Potri.012G091300*) was a newly identified member based on previous study. A large number of *PLL* genes have been identified in many plants, e.g., there are 26 homologous genes in *Arabidopsis*, 12 in rice (*Oryza sativa*) and 46 in the genus *Brassica* (Palusa et al., 2007; Sun and van Nocker, 2010; Jiang et al., 2013b). The 30 *PtPL1* genes were classified into five major groups, and group I was subdivided into four subgroups (Biswal et al., 2014) (**Figure 1**). According to the gene locations on chromosomes, segmental duplications seemed to be the major factors responsible for the expansion of *PtPL1* gene family in *P. trichocarpa* (**Figure 2**). Furthermore, tandem duplications and retro-transposition events have also happened (**Table 1**). On the contrary, only whole genome and chromosomal segmental duplications were found during the evolutionary process of *PLL* gene family in *B. rapa* (Jiang et al., 2013b). Our results also showed that most of the paralogous pairs were under purifying selection pressure in evolution. However, the Ka/Ks value of *PL1-20*/*PL1-12* is 1.4, implying that this gene pairs might experience positive selection after duplication 109.45 mya when *Populus* and *Arabidopsis* lineages diverged (Cao et al., 2016). It could be to improve the adaptability of the organism to the new circumstances. Gene expression data in **Figure 4** further supports this hypothesis: *PL1-12* is specifically expressed in female catkins, male catkins and young leaves, while *PL1-20* obtains higher expression in male catkins and xylem.

### *PtPL1* Genes in *Populus* Participate in Vegetative and Reproductive Processes

Most of PtPL1 proteins were predicted to contain an N-terminal signal peptide (**Figure 1**), which were classified into signal peptide secretary pathway. On the contrary, members from Group V had no predicted signal peptides. PtPL1-2 was predicted to have a chloroplast transit peptide, while no particular sub-cellular localization could be predicted for PtPL1-7 and PtPL1-11. These results were based on bioinformatic prediction. All the 30 *PtPL1* members contain Pec_lyase_C domain (Pfam00544), in spite of some differences in the detailed motifs. Members from subgroup Ia and Ib contain most of the conserved motifs and may perform more comprehensive functions. Members from group II contain the unique Pec_lyase_N domain. Members from group V have the least number of motifs, i.e., absence of signal peptides, the second substrate-binding site, and the cysteine residue for the disulfide bond. The diverse protein structures of different groups may partially contribute to the different expression patterns of *PtPL1s* genes. As shown in the expression heatmap (**Figure 4**), *PtPL1* genes were highly expressed in both vegetative and reproductive tissues, mostly in female catkins, male catkins, xylem and young leaf, and with quite a few in root. Most *PtPL1* genes from subgroup Ia were preferentially expressed in xylem and root, and most members from subgroup Ib were preferentially expressed in xylem, root, and catkins, while the transcripts of genes from subgroup Id, groups II, III, and IV were most abundant in female and/or male catkins. In total, about two thirds of *PtPL1* genes were preferentially expressed in female or male catkins, indicating their important functions in the reproductive developmental process in *Populus*. Previous study had shown that all *PLL* genes were expressed in flowers and several *PLL*s were highly expressed in pollen in *Arabidopsis* (Palusa et al., 2007). Some *PLL* genes in *B. rapa* are expressed in anthers of mature pollen stage and in pistils after pollination. The primary cell wall of the pollen mother cell was degraded by pectinases including *PLL* during meiosis and tetrad stages (Blackmore et al., 2007; Jiang et al., 2013a). Furthermore, *Pectate Lyase-Like10* (*PLL10*) was identified as one of the differentially expressed genes during the late pollen developmental stages and in pistils during the fertilization process in Chinese cabbage (*B. campestris* ssp. *chinensis*) (Jiang et al., 2014). Our main interest is the functions of *PtPL1* genes in wood formation. Wood formation is a sequential developmental process, including complex cell activity (Ye and Zhong, 2015). It has been previously shown that PMEs (EC 3.1.1.11), which catalyze the specific demethylesterification of HGA within plant cell walls, were involved in wood and pollen formation (Pelloux et al., 2007). Prior study showed that the lyases that degrade HG (PL1) were highly expressed in the wood-forming tissues, including tension wood but excluding the wood cell death zone. This suggests a unique role of these lyases in pectin remodeling at the early stages of secondary wall biosynthesis (Geisler-Lee et al., 2006). In this study, the expression profile revealed that *PtPL1-18*, *17*, *19*, *9*, *25*, and *27* have the highest expression level in xylem (**Figure 4**). Among them, *PtPL1-18*, *17*, and *19* are belong to subgroup Ia; *PtPL1-25* and *27* are from subgroup Ib; and *PtPL1-9* is classified into subgroup Ic. It is interesting that five of the six candidate genes are from subgroup Ia and Ib, including the previously published gene *Ptxt1-27.*

### *PtPL1-18* Is Expressed in Primary Xylem and the Cells Undergoing Secondary Wall Thickening

In several previous studies, expression of pectin-related genes were detected in cells undergoing secondary cell wall thickening. A *PL* from *Zinnia elegans* is involved in elongating and differentiating in the development of tracheary elements (Domingo et al., 1998). Several genes involved in pectin biosynthesis and remodeling/degrading were highly expressed in the wood-forming tissues in poplar (Hertzberg et al., 2001; Geisler-Lee et al., 2006). In particular, a poplar PL gene, *Ptxt1-27*, was expressed at the onset of secondary wall formation (Biswal et al., 2014). The vascular tissue of primary growth, develops directly from procambium and is the dominant vasculature in young stem (Dharmawardhana et al., 2010). In this study, *PtPL1-15* was preferentially expressed in IN2 and IN3 suggesting that it might be essential in primary growth of poplar (**Figure 5B**). Recently research indicates the wall plasticity and variations in cell adhesion are key features of the mechanisms involved in wood cell growth. The VC and adjacent radial expansion (RE) zone are the sites of highest expression of genes encoding wall-modifying enzymes, including *PLs* (Mellerowicz and Sundberg, 2008). IN5 and IN 9 have well developed secondary phloem tissue and secondary xylem vessels, as well as fibers with well lignified secondary walls (Dharmawardhana et al., 2010). *PtPL1-17*, *27*, *4* and *25* were all highly expressed in mature stems (**Figures 5C,F–H**); *PtPL1-27* and *25* were preferentially expressed in xylem (**Figures 5F,H**). Meanwhile, *PtPL1-18* and *26* both had two expression peaks, one each in the primary and another was in the secondary growth phases of stem (**Figures 5D,E**). The *PtPL1* genes preferentially expressed in the elongation zone are potential candidates for the control of pectin restructuring during stem elongation. The secondary growth-associated *PtPL1* genes are likely to be involved in pectin remodeling during the early stages of secondary wall biosynthesis and the radial and intrusive growth of cambial derivatives (Dharmawardhana et al., 2010). The expression of *PtPL1-18* was in accordance with the results of its promoter GUS activity assay (**Figure 6**). The *PtPL1-18*-GUS staining was detected in the whole vascular bundle cells of the 4th leaf petioles, in which stage there was no secondary cell walls. In the 7th leaf petioles, the GUS staining reduced in the xylem cell walls which were undergoing secondary cell walls development. In the 12th leaf petioles, GUS staining were absent in mature xylem, nor in the pith, but remained in phloem, pro-cambium and developing xylem. The GUS activity was predominant in the primary xylem of the 4th stems, while relatively less GUS expression was detected in pith and xylem cells undergoing secondary wall formation. The GUS staining in internode 7 was detected in all cell types of the vascular bundle, including the central pith. The GUS staining was the most abundant in the primary xylem, while very little in cortex. In more mature stems, i.e., the 12th internodes, the expression of GUS was much higher in cortex, phloem fibers, phloem, the developing xylem and the primary xylem; on the contrary, the expression was undetectable in the fully lignified cells. The results show that *PtPL1-18* was predominant expressed in parenchyma cells and cells under secondary wall composition. The expression pattern of *PtPL1-18* suggests its unique role in pectin remodeling at the early stages of secondary wall biosynthesis.

### Overexpression of *PtPL1-18* Influences the Onset of Secondary Cell Wall Formation

It is well-known that pectin is very important in primary cell wall development. Recently, some researches also provided evidence of its involvement in secondary wall development (Xiao and Anderson, 2013). Moreover, Dehydrogenated polymers (DHP = coniferyl alcohol polymers = synthetic lignin) interact with pectin to form hydrophobic clusters as monitored by pyrene fluorescence spectroscopy (Lairez et al., 2005). In maize, de-esterified HGA has been shown to form benzyl-uronate crosslinks to lignin (Grabber, 2005). *PME7* in *Eucalyptus pilularis* was primarily associated with cellulose and had an inverse correlation with lignin content (Sexton et al., 2012). Collectively, these observations suggest that different pectin forms could influence the lignification.

In our study, overexpression of *PtPL1-18* in poplar 717 caused decrease of stem height (**Figure 7**), which was similar to that of ectopic overexpression of *pgaII* (a mutated PG gene from *Aspergillus niger*) and overexpression of *PtxtPL1-27* (Lionetti et al., 2010; Biswal et al., 2014). Moreover, collapsed xylem vessel elements with thinner secondary wall were observed in *PtPL1-18* overexpression transgenic trees (**Figure 8**). Several xylan-related mutants named as irregular xylem (irx) due to secondary cell wall deficiencies have been identified in *Arabidopsis*, e.g., both *irx1* and *irx3* had a severe irregular-xylem phenotype (Turner and Somerville, 1997; Brown et al., 2005). Similar phenotypes have also been observed in RNAi lines of *PtrKOR1* gene (*KORRIGAN1* in *P. trichocarpa*), which encodes an endo-1,4-β-glucanase (Yu et al., 2014). The results implied that the secondary wall thickening process in vascular was influenced when *PtPL1-18* was overexpressed. The content of pectin was significantly decreased in the stem tissues of *35S:PtPL1-18* transgenic lines. This should be the result of increased expression of *PtPL1-18* and subsequently faster de-polymerization of pectin, and then increased the content of soluble sugar. However, no obviously consistent changes were observed in the content of cellulose. The content of hemicellulose was increased in Ox-6, Ox-8, and Ox-9 lines. While the lignin content didn’t significantly vary between poplar 717 and the three *35S:PtPL1-18* overexpression lines. Immunolocalization was used to identify the effect of *PtPL1-18* overexpression on modification of pectic polymers. Epitope-specific monoclonal antibodies are important tools to identify diverse polysaccharide in cell walls (Pattathil et al., 2012), in particular pectic polysaccharides including HG, RG-I, and RG-II. In our study, two monoclonal antibodies, JIM5 and CCRC-M35, were used to show the distributions of HG and RGI in stem sections, respectively. JIM5 antibody is particularly important in this study, since PL1 cleave the α-(1,4)-glycosidic bond between the galacturonic acid units of HG and release unsaturated oligogalacturonides. Our results clearly demonstrated that signals of RG-I and HG were dramatically reduced in phloem and developing xylem in the two tested overexpression lines (*PtPL1-18* Ox-6 and Ox-8). Previous studies showed partially methylesterified HGs could be recognized by JIM5, JIM7, and LM20 at the corners between cells in stem of *M. lutarioriparius* stem, whereas RG-I was recognized by LM16 and LM5 in parenchyma cell walls and inner region of secondary cell walls (Cao et al., 2014). JIM5 recognizes partially methylesterified HG. The status of methylation of HG polysaccharide can be affected by both modifying enzymes (e.g., PME) and degradation enzymes (e.g., PL). *Arabidopsis pme35-1* mutant is defective in HG methyl esterification and exhibited a pendant stem phenotype. The signals of JIM5, JIM7, and LM20 monoclonal antibodies signals in the primary cell wall of the cortex and interfascicular fibers were suppressed in the mutant, but lignified cell walls in the interfascicular and xylary fibers were not affected (Hongo et al., 2012). PL treatment of TS tobacco stem internode resulted a decrease of HG signal by JIM5 antibody, indicating effective HG epitope removal (Marcus et al., 2008). In our study, the signal for epitopes of RGI and HG was reduced in phloem and developing xylem of *PtPL1-18* overexpression lines, compared with poplar 717 plants (**Figure 10**). These results suggest that overexpression of *PtPL1-18* contributed to degradation of pectate in phloem and developing xylem. Recent study on *PttPME1* down-regulated lines indicates a high level of HG methylesterification could decrease cellular adhesion and cell wall rigidity and this might trigger mechanosensing responses(Lesniewska et al., 2017). Degradation of pectin during cell expansion by enzymes such as PLs and PGs may enable the incorporation of newly synthesized cell wall components (Marin-Rodriguez et al., 2002). Our results might not fit with expected results from previous work as little influence on the content of lignin by overexpression *PtPL1-18*. The role of pectin in secondary walls still has to be unraveled. On the other hand, the results suggest that indirect changes may still be relevant, such as eliciting structure or solubility changes in non-pectin cell wall components or altering lignin deposition, thus influencing the progress of secondary wall formation.

## Conclusion

Despite its low abundance in the secondary cell walls, recent researches have implied that pectin influences secondary wall formation in addition to its roles in primary wall biosynthesis and modification. In this study, one type of modifying/degrading genes encoding PLs (E.C. 4.2.2.2) were studied in poplar. Geisler-Lee identified *PL1-1* to *PL1-28* in *Populus trichocarpa* and analyzed their expression profile in *Populus*, after that, *PtPL1-29* was identified by Geisler-Lee et al. (2006) and Biswal et al. (2014). *PtPL1-30* was a novel PL1 gene in this study. Through a comprehensive analysis of gene structures, phylogenetic relationships, chromosomal locations, gene duplication, conserved protein motifs, and expression patterns, 30 *PtPL1* genes were characterized. The expression profiles of the *PtPL1* genes provided better understanding in possible functional divergence. Most *PtPL1* genes in subgroup Ia and Ib were highly expressed in vascular tissues, and genes in subgroup Ia were more specifically expressed in xylem. We further showed that *PtPL1-18* from subgroup Ia of the *PtPL1* gene family is preferentially expressed in the primary xylem and the xylem cells undergoing secondary wall composition. When *PtPL1-18* was overexpressed in poplar, plant growth was inhibited and irregular-xylem phenotype was observed, and secondary wall thickening was delayed. Moreover, by analyzing the chemical composition of cell wall, we found that in the wood of *35S:PtPL1-18* transgenic lines, the content of pectin was significantly decreased accompanied by increase of soluble sugar while there was less influence on the major compositions of secondary wall. It was found that pectin epitopes was reduced in phloem and developing xylem cells by immunolocalization. The results provide a visual evidence for the link between pectin modification and secondary wall formation. However, additional evidence based on enzyme complex and enzyme activity analysis will be helpful to establish a clear and direct connection between *PtPL1*-mediated pectin modification and secondary wall formation.

## Author Contributions

BZ, ML, PC, and YB conceived and designed the experiments. YB conducted bioinformatics analysis. YB, DW, FL, and YL performed the experiments. YB, BZ, and PC analyzed the data. YB drafted the manuscript. BZ, ML, and PC edited the manuscript.

## Conflict of Interest Statement

The authors declare that the research was conducted in the absence of any commercial or financial relationships that could be construed as a potential conflict of interest.

## Footnotes

1 http://www.cazy.org

2 http://www.arabidopsis.org/

3 http://genome.jgi.doe.gov/Poptr1_1/Poptr1_1.home.html

4 https://phytozome.jgi.doe.gov/pz/portal.html

5 http://www.softberry.com/berry.phtml?topic=fgenesh\&group=programs\&subgroup=gfind

6 http://smart.embl-heidelberg.de/

7 http://www.cbs.dtu.dk/services/SignalP/

8 http://www.cbs.dtu.dk/services/TargetP/

9 http://web.expasy.org/compute_pi/

10 http://meme-suite.org/tools/meme

11 http://popgenie.org/

12 http://bar.utoronto.ca/efppop/cgi-bin/efpWeb.cgi

13 http://www.affymetrix.com/analysis/index.affx

14 http://web.expasy.org/compute_pi/

15 http://smart.embl-heidelberg.de/

16 https://phytozome.jgi.doe.gov/

17 http://meme-suite.org/tools/meme

## Supplementary Material

**FIGURE S1 |** Multiple sequence alignment of Pec_lyase_C domain in *PtPL1* genes. The brown blocks present Ca^+2^ binding sites; the blue blocks present catalysis sites; the pink block presents disulfide bonds; and the green block presents catalysis sites.

**TABLE S1 |** Gene specific primers used in this study.

**TABLE S2 |** *PtPL1* gene names and gene model IDs in the *Populus* genome assembly v1.1, v2.2, and v3.0.

**TABLE S3 |** Properties of protein products of *PtPL1* genes.

**TABLE S4 |** Prediction of signal peptide and subcellular localization of *PtPL1* genes.

**TABLE S5 |** Probe sets matched to each *PtPL1* gene.
