# Supplementary figures and images for: Characterization and Functional Analysis of the Poplar Pectate Lyase-Like Gene PtPL1-18 Reveal Its Role in the Development of Vascular Tissues
Source: Front Plant Sci. 2017 Jun 28;8:1123. doi: 10.3389/fpls.2017.01123 (PMC5487484; doi:10.3389/fpls.2017.01123)

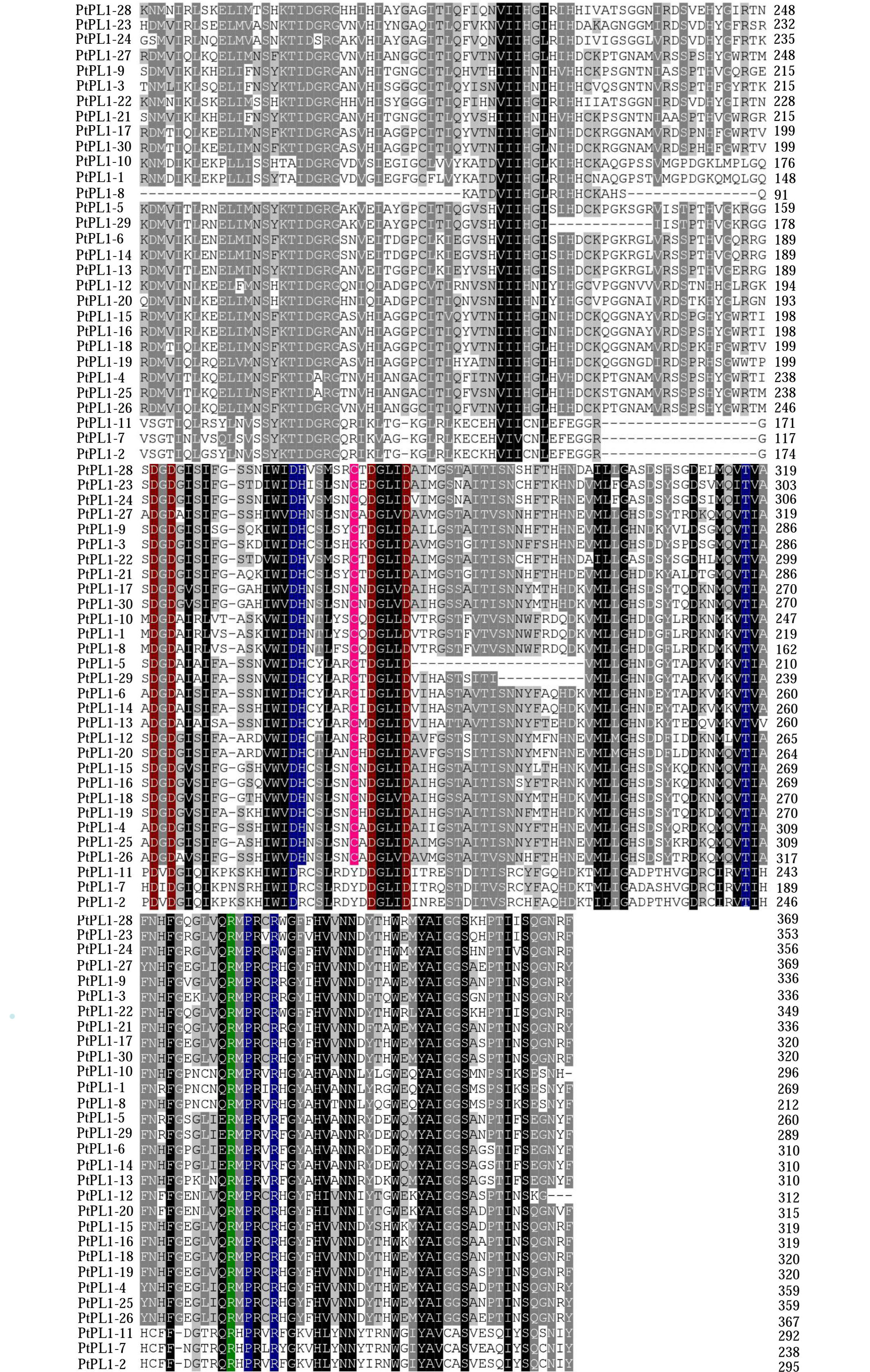

Supplement: Supplementary file 1 [file Image_1.jpg]
