# Supplementary material for: Characterization and Functional Analysis of the Poplar Pectate Lyase-Like Gene PtPL1-18 Reveal Its Role in the Development of Vascular Tissues
Source: Front Plant Sci. 2017 Jun 28;8:1123. doi: 10.3389/fpls.2017.01123 (PMC5487484; doi:10.3389/fpls.2017.01123)
Supplement: Supplementary file 2 [file Table_1.DOCX]

Supplementary Material

**Characterization and functional analysis of the poplar *pectate lyase-like* gene *PtPL1-18* reveal its role in the development of vascular tissues**

**Yun Bai, Dan Wu, Fei Liu, Yuyang Li, Peng Chen, Mengzhu Lu, Bo Zheng^*^**

***** **Correspondence:** Prof. Bo Zheng: bo.zheng@mail.hzau.edu.cn

**Table S1 Gene specific primers**

| Application | Gene Sequence | Primer Forward | Primer Reverse |
| --- | --- | --- | --- |
| **qRT-PCR** | *PtACTIN* | TCATCGGAATGGAAGCTGCTGGTA | TAGTGGAACCACCACTGAGCACAA |
|  | *PtPL1-4* | CATGCGTAACTGGCAATC | AATCCACAAAGGCTCGTC |
|  | *PtPL1-15* | GATTGTAAGCAAGGAGGG | AGATTGTTATGGCTGTGG |
|  | *PtPL1-16* | TGGGAGAAGAATCGGCAAAG | CCATGTCACGAGCGAAAGTG |
|  | *PtPL1-17* | GACTAAACATACACGATTGC | ATCACTATGCCCCAGAAG |
|  | *PtPL1-18* | ACGATTGATGGTAGAGGT | GACAACGAATTATGGTCC |
|  | *PtPL1-19* | TGACTGTAAGCAAGGAGG | GTTGGAGATAGTGATGGC |
|  | *PtPL1-25* | AGACCAGGGACTCTACGC | GCTACGAACCATCGCATT |
|  | *PtPL1-26* | AGGAGGAGACTTGGTTAC | GGTTCACAGGGTCATTAT |
|  | *PtPL1-27* | CCTATTGATGATTGCTGG | TGTCCCTTTTGAACACGA |
| **Sequence amplified** | *PtPL1-18.cds* | TCATGTCTCTCACACCCTTCC | TGTCCCTTTTGAACACGA |
|  | *PtPL1-18.promter* | TAGGTTTATGTCAACGTCCCG | AGAGAGGGAAGGGTGTGAGAG |
|  | *attB* | GGGGACAAGTTTGTACAAAAAAGCAGGCT | GGGGACCACTTTGTACAAGAAAGCTGGGT |
| **RT-PCR** | *PtPL1-18* | CAGCGATTCCTACACTCA | CCTTTCCTGCAACCAAGT |
| **qRT-PCR for Overexpression** | *PtPL1-18* | GGGGCACAGCGATTCCTA | CTCCCTCCAATGGCGTAC |
