# Supplementary material for: Characterization and Functional Analysis of the Poplar Pectate Lyase-Like Gene PtPL1-18 Reveal Its Role in the Development of Vascular Tissues
Source: Front Plant Sci. 2017 Jun 28;8:1123. doi: 10.3389/fpls.2017.01123 (PMC5487484; doi:10.3389/fpls.2017.01123)
Supplement: Supplementary file 3 [file Table_2.DOCX]

Supplementary Material

**Characterization and functional analysis of the poplar *pectate lyase-like* gene *PtPL1-18* reveal its role in the development of vascular tissues**

**Yun Bai, Dan Wu, Fei Liu, Yuyang Li, Peng Chen, Mengzhu Lu, Bo Zheng^*^**

*** Correspondence:** Prof. Bo Zheng: bo.zheng@mail.hzau.edu.cn

**Table S2 *PtPL1* gene names and gene model IDs in the *populus* genome assembly V1.1, V2.2, and V3.0.**

| Gene name | Gene ID |  |  |
| --- | --- | --- | --- |
|  | V1.1 | V2.2 | V3.0 |
| *PtPL1-1* | fgenesh4_pg.C_LG_III001856 | POPTR_0003s21280 | Potri.003G218200 |
| *PtPL1-2* | estExt_fgenesh4_pm.C_LG_VI0607 | POPTR_0006s23090 | Potri.006G214400 |
| *PtPL1-3* | gw1.28.798.1 | POPTR_0006s12390 | Potri.006G122000 |
| *PtPL1-4* | gw1.II.376.1 | POPTR_0002s23990 | Potri.002G238800 |
| *PtPL1-5* | gw1.XI.1367.1 | POPTR_0011s00250 | Potri.T040400 |
| *PtPL1-6* | fgenesh4_pg.C_LG_XI000075 | POPTR_0021s00250 | Potri.011G008100 |
| *PtPL1-7* | estExt_Genewise1_v1.C_LG_VIII1759 | POPTR_0008s04850 | Potri.008G048600 |
| *PtPL1-8* | gw1.V.2503.1 | POPTR_0005s06550 | Potri.005G064500 |
| *PtPL1-9* | gw1.VIII.1321.1 | POPTR_0008s03260 | Potri.008G032700 |
| *PtPL1-10* | fgenesh4_pg.C_LG_I000068 | POPTR_0001s04460 | Potri.001G007400 |
| *PtPL1-11* | fgenesh4_pg.C_LG_XVI000684 | POPTR_0016s08140 | Potri.016G080600 |
| *PtPL1-12* | gw1.I.4759.1 | POPTR_0001s37640 | Potri.001G367800 |
| *PtPL1-13* | gw1.166.38.1 | POPTR_0004s00870 | Potri.004G007300 |
| *PtPL1-14* | gw1.XI.1374.1 | POPTR_0011s00240 | Potri.T040300 |
| *PtPL1-15* | eugene3.00081702 | POPTR_0008s18250 | Potri.008G182200 |
| *PtPL1-16* | eugene3.00100518 | POPTR_0010s06170 | Potri.010G051800 |
| *PtPL1-17* | eugene3.00120841 | POPTR_0012s09310 | Potri.012G091500 |
| *PtPL1-18* | eugene3.00150667 | POPTR_0015s09930 | Potri.015G087800 |
| *PtPL1-19* | estExt_fgenesh4_pg.C_LG_I2215 | POPTR_0001s35960 | Potri.001G339500 |
| *PtPL1-20* | gw1.XI.2918.1 | POPTR_0011s09580 | Potri.011G093400 |
| *PtPL1-21* | gw1.X.2863.1 | POPTR_0010s23570 | Potri.010G229000 |
| *PtPL1-22* | fgenesh4_pg.C_LG_IV001141 | POPTR_0004s12300 | Potri.004G124100 |
| *PtPL1-23* | eugene3.01290005 | POPTR_0015s07590 | Potri.015G064700 |
| *PtPL1-24* | gw1.VIII.2762.1 | POPTR_0008s14810 | Potri.008G148800 |
| *PtPL1-25* | gw1.XIV.4112.1 | POPTR_0014s17620 | Potri.014G178100 |
| *PtPL1-26* | eugene3.00010425 | POPTR_0001s14240 | Potri.001G052300 |
| *PtPL1-27* | estExt_Genewise1_v1.C_LG_III0932 | POPTR_0003s17450 | Potri.003G175900 |
| *PtPL1-28* | gw1.XVII.937.1 | POPTR_0017s11450 | Potri.017G078400 |
| *PtPL1-29* | N.A. | POPTR_0021s00260 | Potri.011G008000 |
| *PtPL1-30* | N.A. | N.A. | Potri.012G091300 |

N.A. represents not available
