# Supplementary material for: Characterization and Functional Analysis of the Poplar Pectate Lyase-Like Gene PtPL1-18 Reveal Its Role in the Development of Vascular Tissues
Source: Front Plant Sci. 2017 Jun 28;8:1123. doi: 10.3389/fpls.2017.01123 (PMC5487484; doi:10.3389/fpls.2017.01123)
Supplement: Supplementary file 4 [file Table_3.DOCX]

Supplementary Material

**Characterization and functional analysis of the poplar *pectate lyase-like* gene *PtPL1-18* reveal its role in the development of vascular tissues**

**Yun Bai, Dan Wu, Fei Liu, Yuyang Li, Peng Chen, Mengzhu Lu, Bo Zheng^*^**

***** **Correspondence:** Prof. Bo Zheng: bo.zheng@mail.hzau.edu.cn

**Table S3 *PtPL1* genes protein products properties**

| Gene symbol | Protein length(aa) | MW (kDa) | pI | Signal P | Pec_lyase_C domain |
| --- | --- | --- | --- | --- | --- |
| *PtPL1-1* | 378 | 42.0 | 9.66 | N.A. | 76-269 |
| *PtPL1-2* | 385 | 42.8 | 7.91 | N.A. | 121-295 |
| *PtPL1-3* | 486 | 53.4 | 5.65 | 1-22 | 143-336 |
| *PtPL1-4* | 442 | 49.2 | 6.67 | 1-24 | 166-359 |
| *PtPL1-5* | 341 | 37.3 | 9.32 | N.A | 87-260 |
| *PtPL1-6* | 393 | 43.1 | 6.36 | 1-18 | 117-310 |
| *PtPL1-7* | 327 | 36.4 | 7.56 | N.A | 64-238 |
| *PtPL1-8* | 394 | 43.7 | 9.67 | 1-25 | 71-212 |
| *PtPL1-9* | 464 | 50.5 | 5.77 | 1-26 | 143-336 |
| *PtPL1-10* | 382 | 42.2 | 9.33 | 1-25 | 104-296 |
| *PtPL1-11* | 386 | 43.1 | 8.62 | N.A. | 118-292 |
| *PtPL1-12* | 266 | 29.5 | 7.66 | 1-19 | 122-312 |
| *PtPL1-13* | 392 | 43.2 | 8.71 | 1-19 | 117-310 |
| *PtPL1-14* | 393 | 43.2 | 6.31 | 1-18 | 117-310 |
| *PtPL1-15* | 402 | 44.0 | 8.21 | 1-21 | 126-319 |
| *PtPL1-16* | 402 | 44.1 | 8.78 | 1-21 | 126-319 |
| *PtPL1-17* | 403 | 44.1 | 6.98 | 1-22 | 127-320 |
| *PtPL1-18* | 403 | 44.1 | 6.82 | 1-22 | 127-320 |
| *PtPL1-19* | 403 | 45.0 | 8.03 | 1-22 | 127-320 |
| *PtPL1-20* | 399 | 44.4 | 5.58 | 1-20 | 121-315 |
| *PtPL1-21* | 496 | 53.7 | 6.23 | N.A | 143-336 |
| *PtPL1-22* | 432 | 48.5 | 9.83 | 1-23 | 156-349 |
| *PtPL1-23* | 435 | 48.5 | 9.35 | 1-24 | 160-353 |
| *PtPL1-24* | 441 | 49.5 | 9.45 | 1-19 | 163-356 |
| *PtPL1-25* | 372 | 41.2 | 8.88 | 1-24 | 166-359 |
| *PtPL1-26* | 450 | 49.3 | 8.44 | 1-24 | 174-367 |
| *PtPL1-27* | 452 | 49.6 | 8.01 | 1-26 | 176-369 |
| *PtPL1-28* | 452 | 50.9 | 9.58 | 1-23 | 176-369 |
| *PtPL1-29* | 370 | 40.5 | 9.39 | 1-25 | 118-289 |
| *PtPL1-30* | 403 | 44.1 | 7.00 | 1-22 | 127-320 |

N.A. represents not available
