# Supplementary material for: Characterization and Functional Analysis of the Poplar Pectate Lyase-Like Gene PtPL1-18 Reveal Its Role in the Development of Vascular Tissues
Source: Front Plant Sci. 2017 Jun 28;8:1123. doi: 10.3389/fpls.2017.01123 (PMC5487484; doi:10.3389/fpls.2017.01123)
Supplement: Supplementary file 5 [file Table_4.DOCX]

Supplementary Material

**Characterization and functional analysis of the poplar *pectate lyase-like* gene *PtPL1-18* reveal its role in the development of vascular tissues**

**Yun Bai, Dan Wu, Fei Liu, Yuyang Li, Peng Chen, Mengzhu Lu, Bo Zheng^*^**

***** **Correspondence:** Prof. Bo Zheng: bo.zheng@mail.hzau.edu.cn

**Table S4 Prediction of signal peptide and subcellular localization**

| Gene symbol | Signal P | Cut off | Target P | RC |
| --- | --- | --- | --- | --- |
| *PtPL1-1* | 1-26. | 0.794 | S | 1 |
| *PtPL1-2* | N.A. | 0.106 | C | 3 |
| *PtPL1-3* | 1-22 | 0.817 | S | 5 |
| *PtPL1-4* | 1-24 | 0.777 | S | 1 |
| *PtPL1-5* | 1-30 | 0.770 | S | 2 |
| *PtPL1-6* | 1-18 | 0.911 | S | 2 |
| *PtPL1-7* | N.A | 0.118 | - | 3 |
| *PtPL1-8* | 1-25 | 0.801 | S | 1 |
| *PtPL1-9* | 1-26 | 0.530 | S | 1 |
| *PtPL1-10* | 1-25 | 0.867 | S | 1 |
| *PtPL1-11* | N.A. | 0.114 | - | 3 |
| *PtPL1-12* | 1-19 | 0.904 | S | 4 |
| *PtPL1-13* | 1-19 | 0.768 | S | 3 |
| *PtPL1-14* | 1-18 | 0.859 | S | 2 |
| *PtPL1-15* | 1-21 | 0.858 | S | 2 |
| *PtPL1-16* | 1-21 | 0.869 | S | 1 |
| *PtPL1-17* | 1-22 | 0.935 | S | 1 |
| *PtPL1-18* | 1-22 | 0.906 | S | 1 |
| *PtPL1-19* | 1-22 | 0.722 | S | 1 |
| *PtPL1-20* | 1-20 | 0.710 | S | 5 |
| *PtPL1-21* | 1-26 | 0.969 | S | 1 |
| *PtPL1-22* | 1-23 | 0.906 | S | 1 |
| *PtPL1-23* | 1-24 | 0.687 | S | 1 |
| *PtPL1-24* | 1-19 | 0.759 | S | 1 |
| *PtPL1-25* | 1-24 | 0.915 | S | 1 |
| *PtPL1-26* | 1-24 | 0.865 | S | 2 |
| *PtPL1-27* | 1-26 | 0.867 | S | 1 |
| *PtPL1-28* | 1-23 | 0.586 | S | 1 |
| *PtPL1-29* | 1-25 | 0.789 | S | 1 |
| *PtPL1-30* | 1-22 | 0.935 | S | 1 |

N.A. represents not available. S signal peptide secretion pathway, C cTP Chloroplast transit peptide, - others. RC, Reliability class, 1: diff > 0.800; 2: 0.800 > diff > 0.600; 3: 0.600 > diff > 0.400; 4: 0.400 > diff > 0.200; 5: 0.200 > diff.
