# Supplementary material for: Characterization and Functional Analysis of the Poplar Pectate Lyase-Like Gene PtPL1-18 Reveal Its Role in the Development of Vascular Tissues
Source: Front Plant Sci. 2017 Jun 28;8:1123. doi: 10.3389/fpls.2017.01123 (PMC5487484; doi:10.3389/fpls.2017.01123)
Supplement: Supplementary file 6 [file Table_5.docx]

Supplementary Material

**Characterization and functional analysis of the poplar *pectate lyase-like* gene *PtPL1-18* reveal its role in the development of vascular tissues**

**Yun Bai, Dan Wu, Fei Liu, Yuyang Li, Peng Chen, Mengzhu Lu, Bo Zheng^*^**

***** **Correspondence:** Prof. Bo Zheng: bo.zheng@mail.hzau.edu.cn

**Table S5 Probe sets matched to each *PtPL1* gene**

| Gene symbol | Probe sets |
| --- | --- |
| *PtPL1-1* | PtpAffx.203635.1.S1_at |
| *PtPL1-2* | PtpAffx.8841.2.A1_at |
| *PtPL1-3* | N.A. |
| *PtPL1-4* | PtpAffx.202640.1.S1_at |
| *PtPL1-5* | PtpAffx.209606.1.S1_at |
| *PtPL1-6* | PtpAffx.209608.1.S1_at |
| *PtPL1-7* | PtpAffx.40050.1.S1_at |
| *PtPL1-8* | PtpAffx.205125.1.S1_at |
| *PtPL1-9* | PtpAffx.207601.1.S1_at |
| *PtPL1-10* | PtpAffx.200040.1.S1_at |
| *PtPL1-11* | PtpAffx.8841.1.A1_at |
| *PtPL1-12* | PtpAffx.201288.1.S1_at |
| *PtPL1-13* | PtpAffx.217351.1.S1_at |
| *PtPL1-14* | PtpAffx.106302.1.A1_at |
| *PtPL1-15* | PtpAffx.208266.1.S1_s_at |
| *PtPL1-16* | N.A. |
| *PtPL1-17* | Ptp.2020.1.S1_s_at |
| *PtPL1-18* | PtpAffx.50265.1.S1_at |
| *PtPL1-19* | Ptp.4652.1.S1_s_at |
| *PtPL1-20* | PtpAffx.209840.1.S1_at |
| *PtPL1-21* | Ptp.4258.1.A1_at |
| *PtPL1-22* | PtpAffx.204147.1.S1_at |
| *PtPL1-23* | PtpAffx.215732.1.S1_at |
| *PtPL1-24* | PtpAffx.208136.1.S1_at |
| *PtPL1-25* | PtpAffx.211973.1.S1_at |
| *PtPL1-26* | PtpAffx.1316.2.S1_s_at |
| *PtPL1-27* | Ptp.4810.1.A1_s_at |
| *PtPL1-28* | PtpAffx.213910.1.S1_at |
| *PtPL1-29* | PtpAffx.209606.1.S1_at |
| *PtPL1-30* | N.A. |

N.A. represents not available
